# Gene therapy for mucopolysaccharidoses: in vivo and ex vivo approaches

**DOI:** 10.1186/s13052-018-0565-y

**Published:** 2018-11-16

**Authors:** Alessandro Fraldi, Marta Serafini, Nicolina Cristina Sorrentino, Bernhard Gentner, Alessandro Aiuti, Maria Ester Bernardo

**Affiliations:** 1 Telethon Institute of Genetic and Medicine (TIGEM), Via Campi Flegrei, 34, Pozzuoli, Naples Italy; 2 Department of Medical and Translational Science, Federico II University, Via Pansini 5, Naples, 80131 Italy; 3 Department of Pediatrics, Dulbecco Telethon Institute, Centro Ricerca M. Tettamanti, University of Milano-Bicocca, Monza, Italy; 4 San Raffaele Telethon Institute for Gene Therapy (SR-TIGET), IRCCS San Raffaele Scientific Institute, Via Olgettina, 60, 20123 Milan, Italy; 5 Unit of Pediatric Immunohematology and Stem Cell Program, IRCCS San Raffaele Scientific Institute, Via Olgettina, 60, 20123 Milan, Italy; 6 Vita Salute San Raffaele University, Milan, Italy

## Abstract

Mucopolysaccharidoses (MPS) are a group of lysosomal storage disorders caused by a deficiency in lysosomal enzymes catalyzing the stepwise degradation of glycosaminoglycans (GAGs). The current therapeutic strategies of enzyme replacement therapy and allogeneic hematopoietic stem cell transplantation have been reported to reduce patient morbidity and to improve their quality of life, but they are associated with persistence of residual disease burden, in particular at the neurocognitive and musculoskeletal levels. This indicates the need for more efficacious treatments capable of effective and rapid enzyme delivery to the affected organs, especially the brain and the skeleton. Gene therapy (GT) strategies aimed at correcting the genetic defect in patient cells could represent a significant improvement for the treatment of MPS when compared with conventional approaches. While in-vivo GT strategies foresee the administration of viral vector particles directly to patients with the aim of providing normal complementary DNA to the affected cells, ex-vivo GT approaches are based on the ex-vivo transduction of patient cells that are subsequently infused back. This review provides insights into the state-of-art accomplishments made with in vivo and ex vivo GT-based approaches in MPS and provide a vision for the future in the medical community.

## Background

Mucopolysaccharidoses (MPS) are a group of 11 lysosomal storage diseases (LSDs) caused by a deficiency of enzymes involved in lysosomal breakdown of the glycosaminoglycans (GAGs)—heparan sulfate (HS), dermatan sulfate (DS), keratan sulfate (KS), chondroitin sulfate (CS), and hyaluronic acid [1, 2]. Consequently, these disorders are associated with the progressive accumulation of GAGs within the blood, connective tissues, and multiple organs. Clinically, MPS are widely heterogeneous and are characterized by a multisystem involvement affecting the liver, spleen, kidney, bone, cartilage, eyes, and central nervous system (CNS) [1, 2]. These diseases are autosomal recessive, except for the X-linked MPS II, and most of them have a variable phenotype from mild to severe. Symptoms develop within the first years of life and, without treatment, patients with the most severe forms typically succumb to disease complications within the first two decades of life. For this reason, timely diagnosis and early therapeutic intervention are crucial to prevent both somatic and CNS pathology.

Current therapeutic approaches for these disorders are directed towards supplying functional enzymes with the goal of lowering GAGs in tissues. These strategies rely on the mechanism of cross-correction [3], which is based on the fact that soluble lysosomal enzymes can be taken up by mannose-6-phosphate receptor-mediated endocytosis into affected cells. At present, enzyme replacement therapy (ERT) and allogeneic hematopoietic stem cell transplantation (HSCT) are the standard treatments for the majority of MPS diseases [4, 5]. Currently, ERT is approved exclusively for MPS I, MPS II, MPS IVA, and MPS VI [6, 7], although the first human treatment with investigational ERT in an MPS VII patient was recently reported [8]. No approved treatment is currently available for the MPS III disorders. Overall, ERT has proven to be effective in controlling most of the visceral manifestations of the diseases, while bone disease, heart valve disease, and corneal opacity have a tendency to be resistant [9]. In particular, CNS involvement has a tendency to be resistant to ERT because of the inability of the lysosomal enzymes to cross the blood–brain barrier (BBB) which impedes the passages of large therapeutic molecules into the brain. Furthermore, ERT has limitations since recombinant enzymes used for ERT can cause immunogenic responses that may translate into lack of efficacy [4, 5]. HSCT was used for the first time for the treatment of an inborn error of metabolism more than 30 years ago in an MPS IH (Hurler syndrome) patient [10]. The principle consists of the possibility of providing the patient with a permanent source of the missing enzyme which is represented by the engrafted donor-derived hematopoietic stem cells (HSCs) and their progeny. As HSCT also enables engraftment of donor-derived microglial cells in the brain, where they can locally produce the enzyme, this treatment, differing from the intravenous administration of enzymes, has the potential to treat CNS manifestations. HSCT has since become the standard of care for MPS IH patients. The availability of international guidelines for allogeneic HSCT in MPS, including high-resolution molecular human leukocyte antigen (HLA) typing for the selection of umbilical cord blood (UCB) units, individualized conditioning regimens, and supportive care, have contributed to render the transplant much safer, with transplant-related mortality < 10% [11, 12]. Overall, allogeneic transplantation is associated with a significant improvement in the survival rate and visceral manifestations, but has also revealed important morbidity due to residual disease burden, in particular at the musculoskeletal and neurocognitive levels [11–15].

Therefore, current transplantation strategies are only partially successful in treating MPS, probably due to the limited efficacy of the protein provided through hematopoiesis which allows only normal enzyme levels to be reached [11, 12]. In addition, the time at which the transplant procedure is performed could be too late to prevent pre-existing or progressive organ damage [16] and, for this reason, the development of safer conditioning regimens that could be employed at earlier ages is a critical accomplishment [17]. Moreover, the advent of new-born screening procedures may prove a major step forward in early identification and treatment of individuals with MPS [18].

Based on these premises, over the last years novel experimental therapies for MPS, including gene therapy (GT), substrate reduction therapy (SRT), anti-inflammatory therapy, and pharmacological chaperone therapy [4, 5, 19], have been investigated. In particular, this review focuses on the scientific evidence demonstrating that GT provides an achievable therapeutic option for MPS disorders. The two main categories of somatic GT consist of 1) the in-vivo infusion of viral vector particles with the aim of transferring normal complementary DNA to the affected cells enabling them to express the missing protein, or 2) the ex-vivo transduction of patient cells that are subsequently infused back. The first form is called in-vivo GT because the vector particles are administered directly to patients. In the ex-vivo procedure, patient cells are cultured in the laboratory, exposed to the viral vector that is carrying the desired gene, and then returned to the patient.

### In-vivo GT approaches

Two important aspects allow MPS to be considered a suitable target for in-vivo GT approaches: monogenic recessive disorders, and that a small amount of therapeutic enzymes is able to improve the somatic and CNS pathology [19]. In-vivo GT is based on the concept of providing a functional copy of the defective gene to slow or reverse the disease state. A variety of different viral vectors, such as adenovirus, adeno-associated virus (AAV), retrovirus (RV), and lentivirus (LV), have undergone preclinical and clinical testing for their ability to mediate phenotypic improvements following their systemic, CNS direct, and intra-cerebrospinal fluid (CSF) injections in MPS animal models [4, 19, 20].

**Fig. 1:**
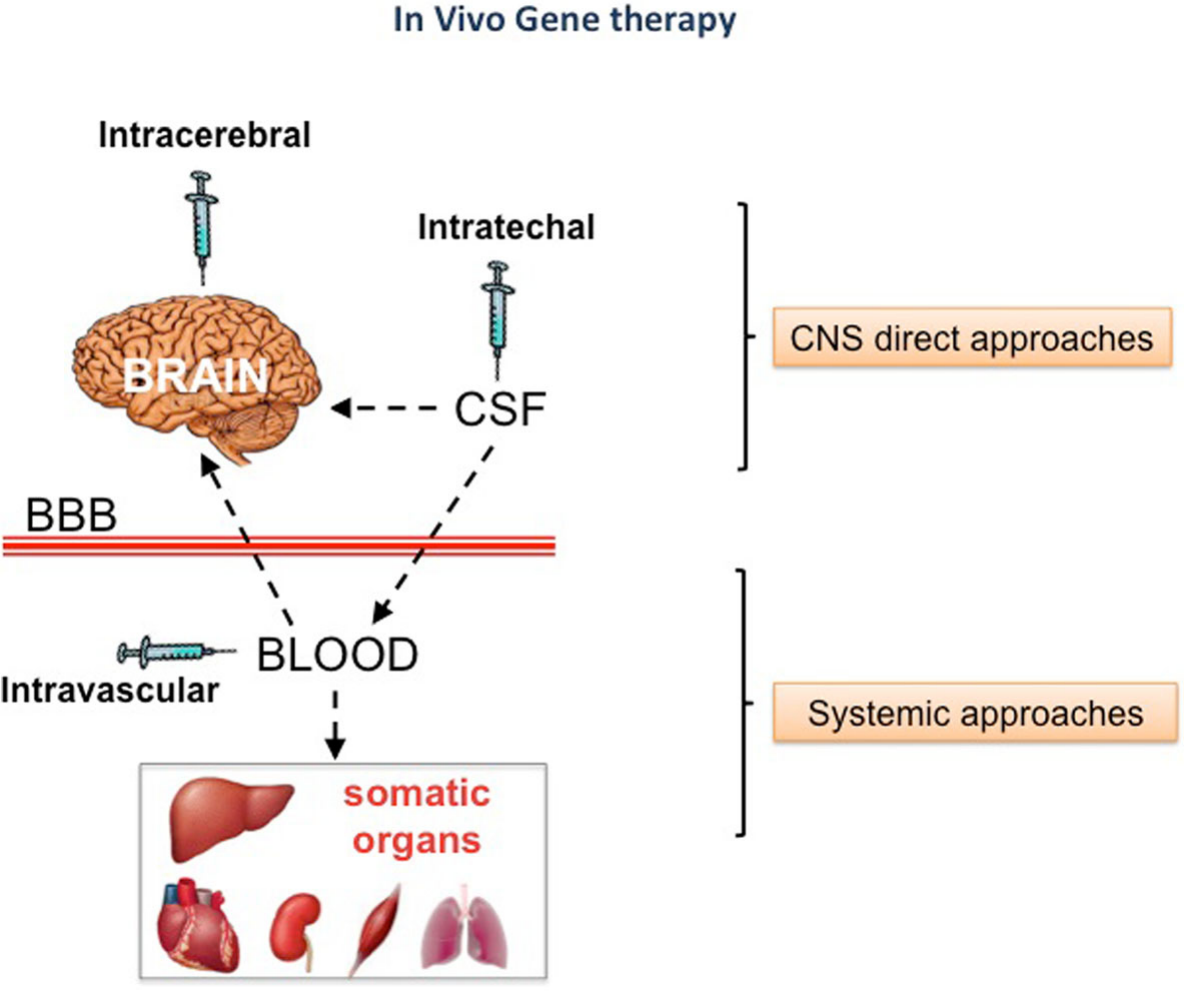
Delivery routes in in-vivo GT approaches for MPS. Approaches for in-vivo GT of MPS may employ two different routes of viral vector administration. In direct central nervous system (CNS) administration routes, the viral vectors access the CNS via parenchyma (intracerebral injections) or via cerebrospinal fluid (CSF) (intrathecal/intraventricular injections). CNS is primarily targeted by these approaches. However, leakage of vectors in the bloodstream may result in the targeting and correction of somatic organ pathology. CNS direct approaches are generally invasive for clinical purposes. The second group of approaches is instead based on systemic injections (often directly into the blood stream) of viral vectors. These approaches efficiently target somatic organs and tissues. However, different strategies may be used to make these approaches capable of overcoming the blood–brain barrier (BBB) obstacle and treat the brain. Systemic approaches are minimally invasive and, in principle, more suitable for clinical purposes

Among viral vectors for gene replacement, AAV vectors are those most used for in-vivo gene transfer since they are safe, provide significantly long transgene expression, and may be generated with variable serotypes allowing efficient delivery of therapeutic genes to different target tissues [21, 22]. Therefore, here, we will review mainly in-vivo GT studies based on AAV-mediated gene delivery. Viral vectors may be delivered using different administration routes (Fig. 1). Direct administration to the CNS represents a suitable delivery strategy when the CNS is the major therapeutic target. Direct CNS delivery may be achieved by either intracerebral or intrathecal/intraventricular injection in which the therapeutics access the brain, respectively, via parenchyma or intra-CSF. Intra-CSF delivery may also target somatic organs due to leakage of viral vectors in the blood stream. Direct CNS-targeting approaches generally represent high-invasive approaches for human therapeutic application. Systemic injection via the intravascular route is a noninvasive delivery strategy that represents the elective way to reach peripheral organs and tissues. Nevertheless, since every neuron in the brain is perfused by its own blood vessel, the intravenous administration of the therapeutic molecule can virtually reach all brain regions [23]. However, the BBB is not permeable to all the molecules and might impede effective delivery of therapeutic agents [24]. Thus, in general, while the somatic organs are efficiently targeted by a therapeutic molecule upon intravascular administration, the CNS instead is little or not targeted. However, as described below, different strategies may be employed to make intravascular-based delivery routes capable of overcoming the BBB obstacle.

#### Intracerebral injection

AAV viral vector administration in a specific area of the brain parenchyma has been extensively used in small and large animal models of MPS I [25], MPS IIIA [26], MPS IIIB [27], and MPS VII [28], where this strategy was able to rescue primary and secondary storage, thus preventing functional deficits. The promising results obtained in the preclinical studies in an MPS IIIA mouse model led to a phase I/II clinical trial in MPS IIIA patients (Lysogene) [29]. Four children received intracerebral injections of an AAV vector serotype rh.10-SGSH-IRES-SUMF1 delivered bilaterally to the white matter anterior, medial, and posterior to the basal ganglia at two different depths in a stereotaxic device at a dose of 7.2 × 10^11^ viral genomes/patient. Before treatment, all children were able to walk, but their cognitive abilities were abnormal and had declined; three out of four patients showed brain atrophy. The procedure was well tolerated with an absence of adverse events related to the injected product. At 1-year follow-up, neuropsychological evaluations suggested a possible, although moderate, improvement in behavior, attention, and sleep in three patients, whereas brain atrophy was stable in two patients but progressed in the others (Table 1) [29]. The high invasiveness of the surgical procedure and the limited distribution of the lysosomal enzymes to the injection site represent the major drawbacks of this approach. A phase II/III clinical trial based on the multisite delivery of AAV rh.10 encoding only *N*-sulfoglucosamine sulfohydrolase (SGSH) has been planned by Lysogene.

**Table 1:** Clinical trials of ex-vivo and in-vivo gene therapy (GT) in mucopolysaccharidosis (MPS)

Disease	Study phase	Type of vector	Route	No. of treated patients	Outcome	Sponsor
In vivo						
MPS IIIA	I/II	AAVrh.10-SGSH-IRES-SUMF1	IC	4	Improvement in behaviour; brain atrophy stable/worse	Lysogene
MPS IIIA	II/III	AAVrh.10-SGSH	IC	In preparation	–	Lysogene
MPS IIIA	I/II	AAV9-SGSH	Intra-CSF	Starting enrolment	–	ESTEVE & UAB
MPS IIIA	I/II	AAV9-SGSH	Systemic	Starting enrolment	–	Abeona Therapeutics
MPS IIIB	I/II	AAV9-NAGLU	Intra-CSF	In preparation	–	ESTEVE & UAB
MPS IIIB	I/II	AAV9-NAGLU	Systemic	In preparation	–	Abeona Therapeutics
MPS I	I/II	AAV9-SGSH	Intra-CSF	In preparation	–	REGENXBIO
MPS I	I/II	AAV-ZFN	Systemic	Starting enrolment (for attenuated form)	–	Sangamo Therapeutics
MPS II	I/II	AAV9-SGSH	Intra-CSF	In preparation	–	ESTEVE & UAB; REGENXBIO
MPS II	I/II	AAV-ZFN	Systemic	Starting enrolment (for attenuated form)	–	Sangamo Therapeutics
MPS VI	I/II	AAV8-ARSB	Systemic	Starting enrolment	–	Fondazione Telethon
Ex vivo						
MPS IH	I/II	LV	IV	In preparation	–	Fondazione Telethon
MPS IIIA	I/II	LV	IV	In preparation	–	Orchard Therapeutics

*AAV* adeno-associated virus, *IC* intracerebral injection, *intra-CSF* intra-cerebrospinal fluid, *IV* intravenous, *LV* lentiviral vector, *UAB* University Autonoma de Barcelona

#### Intra-CSF injection

The intra-CSF AAV vector administration was recently proven to be efficacious in correcting somatic and CNS pathology in different MPS disorders in preclinical models. The main advantage of this strategy is the possibility of injecting the vector in the ventricles (i.e., lateral ventricle administration) or subarachnoid spaces (i.e., cisterna magna and intrathecal lumbar injections) filled with the CSF, thus reaching all the CNS structures surrounded by the CSF. Moreover, the leakage into the bloodstream makes these approaches capable of also targeting somatic tissues. Different examples of intra-CSF injections report the widespread CNS transduction and rescue of phenotypic aspects in MPS I [30], MPS IIIA [31, 32], MPS IIIB [33], and MPS VII [34] small and large animal models using different AAV serotypes. Among the AAV serotypes tested, the serotype 9 has been shown to be the most promising [35]. This, together with the safety of the lateral ventricle surgical procedure has recently led to approval of a clinical trial based on AAV9-mediated injection into the lateral ventricles of MPS IIIA patients (ESTEVE and Universitat Autònoma de Barcelona (UAB)) (Table 1). Clinical trials based on similar approaches are planned for MPS IIIB, MPS I, and MPS II (ESTEVE, UAB, and REGENXBIO).

However, intra-CSF-based approaches have some disadvantages, such as limited long-term gene expression and toxicity, factors that have to be considered before translating therapeutic strategies based on intra-CSF injection into clinical protocols.

#### Systemic administration

The intravenous liver-targeting strategy represents a noninvasive GT approach for the treatment of MPS. AAV vector serotypes with high tropism to the liver and liver-specific promoters have been tested to target the liver and convert it into a “factory” organ for the production and secretion of supraphysiological amounts of the lysosomal enzymes. The secreted enzymes traveling through the blood circulation reach the peripheral affected organs and are trafficked to the lysosomes. These approaches were successfully employed for the treatment of somatic pathology in MPS I and MPS VI animal models [36, 37], and a phase I/II clinical trial is currently ongoing for MPS VI patients (The MeuSIX consortium)**.** This trial is based on AAV vector serotype 8 with liver-specific thyroxine-binding globulin (TBG) promoter driving the expression of the human ARSB gene. Vectors will be administered into a peripheral vein at three different doses (low dose, 2 × 10^11^ gc/kg; intermediate dose, 6 × 10^11^ gc/kg; high dose, 2 × 10^12^ gc/kg). However, the presence of the BBB makes liver-targeting strategies less suitable for the treatment of neuropathology in MPS. Indeed, intravenous administration of a lysosomal enzyme is able to markedly decrease the abnormal storage in non-neurological tissue, but has no or little effect on CNS pathology in disease mouse models [38]. The development of systemic therapeutic strategies that can overcome the major obstacle posed by the BBB represents the real challenge to establishing efficacious and safe therapies for MPS involving the CNS [39]. An approach to cross the BBB is to modify the therapeutic enzymes so that they can access one of the physiological transport systems localized within the BBB responsible for the delivery of molecules from the blood to the brain. Over the last years, a number of strategies have been developed to engineer the lysosomal enzymes with BBB-targeting motifs. These modified versions of the enzymes have been used in preclinical studies in MPS IIIA, MPS IIIB, MPS VII, MPS I, and MPS II animal models [39, 40]. In MPS IIIA, for example, two different strategies have been used to engineer the sulfamidase (the enzyme defective in MPS IIIA). In one, the binding domain of the apolipoprotein B, a protein that is normally transported across the BBB by low-density lipoprotein receptor (LDLR) binding, was fused to the C-terminal of the sulfamidase to allow the enzyme to be transcytosed through the BBB using the LDLRs on the BBB as a port to entry the brain. AAV vector serotype 8 (this serotype displays high liver tropism) encoding the chimeric sulfamidase was used as a vehicle to selectively target the liver via intravascular injection and convert it into a factory organ to produce the chimeric enzyme. Moreover, to increase the secretion of the sulfamidase form in the liver, the enzyme was further modified at the N-terminal with an alternative signal peptide derived from the highly secreted protein iduronate-2-sulfatase. This approach was successful in reverting neuropathology in MPS IIIA mice [41]. In a second approach, a BBB-penetrating form of sulfamidase was produced by re-engineering the enzyme as an IgG fusion protein, where the IgG domain is a monoclonal antibody (mAb) against the human insulin receptor (HIR). The HIRmAb domain of the HIRmAb-SGSH fusion protein acts as a molecular “Trojan horse” to ferry the fused enzyme across the BBB [42].

New attractive systemic AAV-mediated gene therapeutic approaches for the treatment of neuropathology are based on the use of specific AAV serotypes (e.g., AAV9, AAVrh10, AAVrh8) that are able to cross the BBB and target the CNS [43, 44]. A clinical trial based on AAV9 delivered intravascularly is currently under evaluation for MPS IIIA and is planned for MPS IIIB (Abeona Therapeutics). Preliminary data are now available for the first MPS IIIA patients treated with AAV9-SGSH. These data show a dose- and time-dependent CNS HS reduction together with neurocognitive benefit in the higher bar (older, low-dose; cohort 1), which implies benefit could be superior in cohort 2 (younger, high-dose).

In summary, AAV-mediated in-vivo GT for MPS is rapidly advancing towards the development of clinical protocols. However, some aspects, such as dosage of the viral vectors and their potential toxicity, have to be thoroughly understood before routinely applying these strategies for clinical management of patients. Another important field of investigation in in-vivo GT of genetic diseases is the possibility of directly modify defective genes by site-specific in vivo genome editing. Genome editing mediated by zinc finger nucleases (ZFN), transcription activator-like effector, or CRISPR/Cas9 nucleases have been explored for a number of genetic diseases at both the preclinical and clinical level [45]. Therapeutic strategies based on targeted insertion of the functional gene at the albumin locus in hepatocytes through in-vivo ZFN-mediated genome editing are currently being explored for attenuated forms of MPS I and MPS II (Sangamo BioSciences) and it is likely that these strategies will broaden clinical application in the near future.

### Ex-vivo GT approaches

**Fig. 2:**
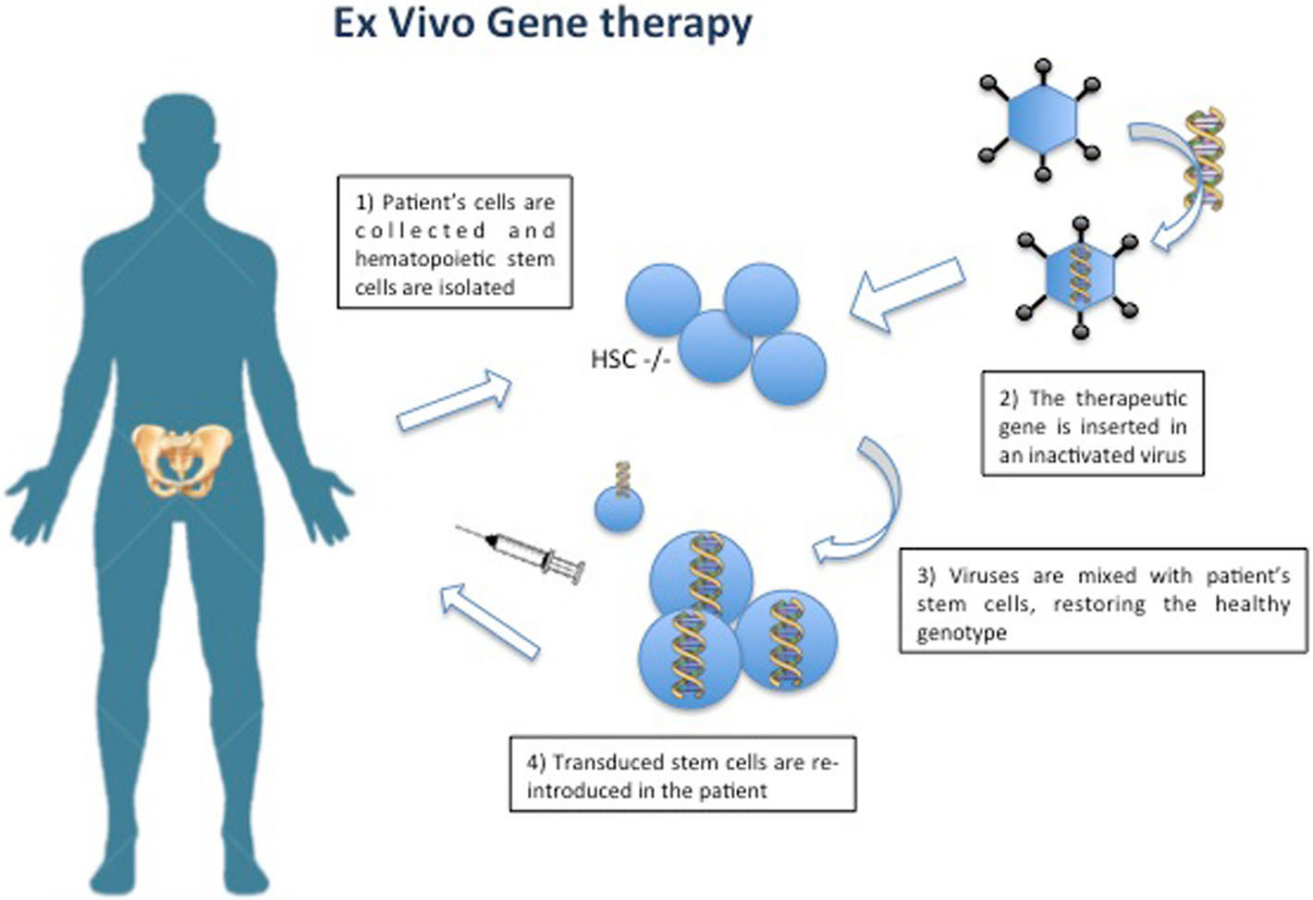
Ex-vivo GT approach for MPS. In ex-vivo GT approaches, patient cells are collected and stem cells are isolated; thereafter, they are mixed with the viral vector in which the therapeutic gene has been inserted. The final transduced stem cells are later re-infused in the patient after administration of a conditioning regimen and, by engrafting in the patient tissues, are able to restore the healthy phenotype. Modified from Penati et al., J Inherit Metab Dis. 2017, in press

Ex-vivo GT entails the procurement and purification of target cells (i.e., CD34^+^ HSCs in the case of HSC-GT) that are transduced in vitro with a viral vector to express the therapeutic gene. Subsequently, the gene-corrected cells are returned to the patient after a chemotherapy-based preparative regimen of various intensity according to the disease treated [46], where they can engraft and restore the healthy phenotype [47–50] (Fig. 2). Both retroviral (RV) and lentiviral (LV) vectors that are able to integrate into the host genome can be employed to correct the altered gene in the HSCs and in their progeny. It has been demonstrated that the use of self-inactivating LVs is associated with a lower risk of insertional mutagenesis, and no events of leukemic transformation have been reported so far in patients treated in HSC-GT trials with this approach [51]. Moreover, LVs have the advantage of infecting nondividing cells and permitting higher levels of gene marking [52].

Ex-vivo GT strategies have been tested in animal models and in clinical trials in LSDs, and in particular in MPS. RV-based HSC-GT proved to be effective in restoring enzyme activity and providing therapeutic benefit to visceral organs in MPS I mice; however, the CNS pathology was not sufficiently corrected, likely due to insufficient enzyme production by the engineered cells [53]. An LV-based approach has been employed to express alpha-l-iduronidase (IDUA) in an erythroid-specific manner by the use of a lineage-specific promoter in an MPS I mouse model. While gene-modified erythrocytes effectively released the enzyme into the circulation which was then distributed to the affected tissues, the neurological outcome was not improved compared with allogeneic HSCT [54]. The possibility of preventing and correcting neurological manifestations in mouse models of LSD by transplanting LV-transduced HSCs has also been reported for metachromatic leukodystrophy (MLD) and globoid cell leukodystrophy (GLD) [55–57]. Using this approach, microglia replacement by gene-corrected cells provided a population of efficient scavenger cells within the CNS with the potential to clear the accumulated substrate and which represents a new and effective source of bioavailable enzyme in the brain [55, 56]. In particular, in the mouse model of GLD, the infusion of HSCs corrected with an LV vector encoding for the galactocerebrosidase (GALC) gene was associated with reconstitution of the missing enzymatic activity in the liver and brain, together with improved survival and amelioration of the disease phenotype in the affected animals [57]. In the MLD mouse model, LV-based HSC-GT, but not transplantation of wild-type (WT) HSCs, was associated with prevention and correction of the neurological manifestations [55, 56]. In the MPS II mouse model, an LV-based HSC-GT approach was able to correct and prevent neuronal manifestations by ameliorating lysosomal storage and autophagic dysfunction in the brains of MPS II mice [58]. After transplantation of LV-transduced HSCs, increased enzyme activity was measured both in visceral organs and the CNS compared with naive mice, together with decreased levels of GAGs.

Based on these preclinical data, the therapeutic potential of LV-based HSC-GT has been tested in patients affected by MLD [49, 59] with the rationale to express supranormal levels of the therapeutic enzyme by gene-modified HSCs, particularly at the level of organs that are more difficult to reach, i.e., the CNS. The results of the first nine treated patients within a phase I/II study showed stable engraftment of the transduced HSCs in the bone marrow (BM) and peripheral blood of patients resulting in supranormal ARSA activity in hematopoietic lineages and in the CSF [49, 59]. The presence of ARSA activity in the CSF represents indirect evidence that HSC-derived cells have migrated to the brain and produced the enzyme locally. These findings were associated with a substantial therapeutic benefit of the procedure; better results were obtained in children who were treated when pre- or very early symptomatic. These data provide the first formal proof in humans that HSC-GT could represent a valuable therapeutic option for LSDs and constitutes an interesting platform for testing similar strategies in MPS disorders.

Indeed, published data on MPS IH patients treated with ERT and/or allogeneic HSCT indicate the need for a more efficacious treatment capable of early and higher levels of delivery to the affected organs, especially the brain and the skeleton. Indeed, allogeneic HSCT of MPS IH patients has been reported to significantly reduce patient morbidity and to improve their quality of life, but the therapeutic effect on bone and CNS lesions remains limited [15]. The musculoskeletal manifestations often require orthopedic surgical interventions after allo-HSCT and still deteriorate and impact the quality of life in most transplanted patients, probably due to the limited penetration of the expressed enzyme into the tissues [12, 14].

According to these premises, and taking advantage of the technology and expertise gained in the context of MLD, LV-driven supranormal IDUA reconstitution in HSCs and their progeny was tested at SR-TIGET with the aim of improving the outcome of the neurological and skeletal manifestations in MPS I mice. Therefore, MPS I mice were challenged with the transplantation of WT and LV-transduced *Idua*^*−/−*^ HSCs in which enzymatic activity was restored to supranormal levels [60]. Results showed robust and effective delivery of the functional IDUA enzyme to diseased tissues, including the CNS where supranormal enzymatic activity was measured. This finding is particularly relevant in light of the inability of transplanted HSCs from normal WT donors (WT HSCT) to deliver comparable amounts of enzyme to the brain. Metabolic correction of the affected tissues was also demonstrated, as shown by the clearance of accumulated GAGs within hematopoietic and nonhematopoietic cells; this finding suggests the occurrence of active secretion of the functional enzyme by the gene-corrected progeny of the transplanted cells and its reuptake by the resident populations. A long-term toxicology/genotoxicity study was also conducted in MPS I mice under Good Laboratory Practice (GLP) conditions, employing an IDUA-LV produced under large-scale clinical grade conditions and by performing secondary transplantation into MPS I mice [61]. The study confirmed the long-term efficacy and safety of the approach in a rigorous testing environment. Indeed, stable engraftment of gene-modified cells was found, as well as sustained transgene expression resulting in the reconstitution of the defective IDUA activity at above WT levels in most of the GT-treated mice. Moreover, lack of evidence of any toxic or tumorigenic potential of IDUA-LV-transduced murine HSCs in primary and secondary transplant recipients was demonstrated. In a biodistribution study performed under GLP conditions, the repopulation of immunodeficient NSG mice by human UCB-derived CD34^+^ cells transduced with IDUA-LV was tested and compared with mock-transduced cells. Results confirmed stable IDUA overexpression in the human graft and in the myeloid progeny, as well as no functional impairment of human HSCs overexpressing IDUA, and the absence of recombinant replication-competent LV [61]. Importantly, BM CD34^+^ cells from MPS I patients behaved similar to those obtained from volunteer donors in terms of transducibility by IDUA-LV and reconstitution and overexpression of IDUA activity, as well as with regards to their clonogenic potential and capacity to repopulate immunodeficient mice [61].

Altogether, the significant therapeutic benefit observed in MLD patients and in MPS I mice and the data on the safety and tolerability of the approach both in mice and humans support the clinical testing of HSC-GT in MPS IH patients with the goal of augmenting the benefit of allogeneic HSCT, especially at the levels of the bone and CNS. Moreover, compared with allograft, the autologous procedure is associated with a reduced transplantation-related morbidity and mortality and, in particular, avoids the risks of allo-immune complications, i.e., rejection and graft-versus-host disease (GVHD). A phase I/II clinical trial is being implemented at SR-TIGET with the aim of testing the feasibility and safety of HSC-GT in MPS IH patients (Table 1). The trial will aim to achieve supranormal IDUA enzyme levels in patient blood cells to improve and/or prevent damage in visceral organs, CNS, and the skeleton. Concerning the skeletal tissue, the possibility that expression of supranormal enzyme levels might also favor the cross-correction of bone resident cells, such as mesenchymal stromal cells and osteoclasts, which have been reported to play a role in the skeletal manifestations of the disease, will be also investigated [62, 63].

MPS IIIA, an LSD caused by mutations in SGSH and resulting in HS accumulation and progressive neurodegeneration, is another potentially appealing target for which an LV-based, ex-vivo HSC-GT approach, similar to MPS IH, has been developed. Initial preclinical studies showed neurological improvement in the mouse model only when LV gene transfer was coupled to transplantation of WT cells. Subsequently, the use of an LV encoding for a codon-optimized therapeutic gene (SGSH) under the control of a myeloid-specific promoter to improve brain expression via monocyte/microglial specificity led to transgene overexpression and disease correction. In particular, the myeloid-specific promoter CD11b gave significantly higher monocyte/B-cell expression than PGK or CD18 promoters. When autologous MPS IIIA HSCs transduced with either LV-PGK-coSGSH or LV-CD11b-coSGSH vectors expressing codon-optimized SGSH were transplanted into MPS IIIA mice, the use of the latter vector was associated with significantly higher brain expression, normalization of MPS IIIA behavior, brain HS, and neuroinflammation [64]. The use of the myeloid-specific promoter CD11b to control the expression of the SGSH gene represents the main difference in this approach as compared with the strategy proposed for MPS IH patients.

A phase I/II clinical trial of HSC-GT for MPS IIIA, sponsored by Orchard Therapeutics Ltd., is planned for 2018 (Table 1). Enrolled patients will undergo a conditioning regimen based on myeloablative doses of busulfan, followed by infusion of autologous HSCs transduced with an LV vector expressing SGSH under the control of the CD11b myeloid-specific promoter. Safety and efficacy of the administration of the genetically modified HSCs will be evaluated.

In summary, over the last few years substantial progress has been made in HSC-GT for LSDs. Preclinical studies based on ex-vivo approaches for MLD, MPS IH, and GLD have shown that these strategies may have therapeutic impact on disease outcome. Moreover, a phase I/II LV-mediated HSC-GT protocol for MLD has suggested clinical benefit in late infantile presymptomatic patients. Ongoing and future preclinical and clinical trials will provide essential insights into vector design, disease correction at specific target organs, and the possibility of employing less toxic conditioning regimens to allow for the treatment and possible cure of LSDs through gene therapy approaches.

## Conclusion

Because current therapeutic strategies are only partially successful in curing MPS, novel therapeutic approaches based on GT are being developed for various forms of these diseases. While preclinical studies have already demonstrated the potential benefit of both in-vivo and ex-vivo GT approaches in several MPS mouse models, phase I/II clinical trials are under development for various MPS. These strategies are being implemented with the rationale of providing high levels of the therapeutic enzyme to the patient either by direct infusion of the viral vector or by the engrafted gene-modified HSCs. This will hopefully allow for superior correction also at the level of organs that are more difficult to reach, such as the skeleton and the CNS. The development of strategies that allow us to overcome the BBB obstacle using in-vivo GT approaches with systemic injection is another challenge for the near future. The possibility of directly modifying defective genes by site-specific in-vivo genome editing is also being explored for a number of genetic diseases, including MPS.

The implementation of neonatal screening, which allows for early diagnosis, is of critical importance to ensure timely treatment of congenital disorders such as MPS IH in which a younger age at transplantation together with preservation of cognitive function have been reported as major predictors for superior cognitive development after allogeneic HSCT. Neonatal screening programs have already been developed in the US for SCID and some LSD, and pilot studies are on-going also in Italy in selected regions for ADA-SCID and MPS-IH [18, 65–67]. Thanks to the implementation of neonatal screening, and considering that UCB has become the preferential HSC source for allogeneic HSCT in LSD and in particular in MPS IH both in the US and Europe, UCB may also be considered in the future as an alternative source for HSC-GT in newborn/toddlers after dedicated storage of autologous UCB units. In this regard, strategies aimed at the expansion and improvement of transduction of primitive HSCs could expand the field of application of ex-vivo gene therapy [68]. The development of conditioning regimens associated with low extramedullary toxicity, such as those based on monoclonal antibodies selectively depleting blood cells in the BM [17], might open new frontiers to increase the application of HSC-GT at neonatal age.

## Abbreviations

AAV: Adeno-associated virus
BBB: Blood–brain barrier
BM: Bone marrow
CNS: Central nervous system
CSF: Cerebrospinal fluid
ERT: Enzyme replacement therapy
GAG: Glycosaminoglycan
GLD: Globoid cell leukodystrophy
GLP: Good Laboratory Practice
GT: Gene therapy
HIR: Human insulin receptor
HS: Heparan sulfate
HSC: Hematopoietic stem cell
HSCT: Hematopoietic stem cell transplantation
IDUA: Alpha-l-iduronidase
LDLR: Low-density lipoprotein receptor
LSD: Lysosomal storage disease
LV: Lentivirus(al)
mAb: Monoclonal antibody
MLD: Metachromatic leukodystrophy
MPS: Mucopolysaccharidosi(e)s
RV: Retrovirus(al)
SGSH: *N*-sulfoglucosamine sulfohydrolase
UCB: Umbilical cord blood
WT: Wild-type
ZFN: Zinc finger nucleases
